# A natural cortical axis connecting the outside and inside of the human brain

**DOI:** 10.1162/netn_a_00256

**Published:** 2022-10-01

**Authors:** Claus C. Hilgetag, Alexandros Goulas, Jean-Pierre Changeux

**Affiliations:** Institute of Computational Neuroscience, University Medical Center Eppendorf, Hamburg University, Hamburg, Germany; Department of Health Sciences, Boston University, Boston, MA, USA; CNRS UMR 3571, Institut Pasteur, Paris, France; Communications Cellulaires, Collège de France, Paris, France

**Keywords:** Comparative connectomics, Connectomic hypothesis, Hominization, Brain evolution, Cortical development

## Abstract

What structural and connectivity features of the human brain help to explain the extraordinary human cognitive abilities? We recently proposed a set of relevant connectomic fundamentals, some of which arise from the size scaling of the human brain relative to other primate brains, while others of these fundamentals may be uniquely human. In particular, we suggested that the remarkable increase of the size of the human brain due to its prolonged prenatal development has brought with it an increased sparsification, hierarchical modularization, as well as increased depth and cytoarchitectonic differentiation of brain networks. These characteristic features are complemented by a shift of projection origins to the upper layers of many cortical areas as well as the significantly prolonged postnatal development and plasticity of the upper cortical layers. Another fundamental aspect of cortical organization that has emerged in recent research is the alignment of diverse features of evolution, development, cytoarchitectonics, function, and plasticity along a principal, natural cortical axis from sensory (“outside”) to association (“inside”) areas. Here we highlight how this natural axis is integrated in the characteristic organization of the human brain. In particular, the human brain displays a developmental expansion of outside areas and a stretching of the natural axis such that outside areas are more widely separated from each other and from inside areas than in other species. We outline some functional implications of this characteristic arrangement.

## Author Summary

Which brain connectivity features underlie the extraordinary human cognitive abilities? We have proposed a set of relevant connectomic fundamentals, including the increased sparsification, hierarchical modularization, as well as increased depth and cytoarchitectonic differentiation of cortical networks due to the prolonged prenatal development and size increase of the human brain. Such features are complemented by a shift of projection origins to the upper layers of many cortical areas and significantly prolonged postnatal development of such layers. Another fundamental aspect of cortical organization is the alignment of diverse features of evolution, development, cytoarchitectonics, function, and plasticity along a natural cortical axis from sensory (“outside”) to association (“inside”) areas. Here we highlight how this natural axis is integrated in the characteristic connectivity organization of the human brain.

## CONNECTOMIC FUNDAMENTALS OF THE HUMAN BRAIN

The extraordinary cognitive abilities of modern humans, which have arisen over an evolutionarily short time span of less than one million years, present an apparent paradox, as there are no conspicuous differences in the size or the content of the human genome relative to nonhuman primates that could readily explain these abilities. Moreover, no singular genes have been identified that are specifically linked to human cognitive abilities such as language, which rather appear to arise from the interactions of a multitude of genes. As an alternative, we have recently put forward a connectomic hypothesis for the hominization of the brain (Changeux et al., 2021). This hypothesis rests on the assumption that primarily small changes in gene expression, rather than genome content, have affected the development of the human brain and with it its connectivity, as an intermediate functional phenotype (Changeux, 2017).

In particular, many human connectomic features might be accounted for by extended prenatal development and the resulting substantial increase in brain size within the global neural organization of the primate brain (Buckner & Krienen, 2013; Nieuwenhuys & Puelles, 2016). Such increased brain size, in turn, leads to a larger number of cortical neurons as well as areas, and the sparsification and increased hierarchical (encapsulated) modularity of cortical connections. The combination of these features with the developmental expansion of upper cortical layers, the increased laminar differentiation of cortical projections, as well as a prolonged postnatal brain development, which provides a substantially expanded exposure to nongenetic interactions with the physical, social, and cultural environment, gives rise to categorically human-specific cognitive abilities and particularly language.

In this hypothesis, we distinguish characteristic aspects of two kinds, specifically, features of the human brain that are scaled within a primate envelope of brain organization versus features that are uniquely human. Concretely, we consider as scaled features the developmental expansion of the human brain with the resulting sparsification and hierarchical modularization of its connectivity as well as expanded depth and cytoarchitectonic specialization of cortical processing stages, in particular, the cytoarchitectonic differentiation between the core and periphery of cortical connectivity. By contrast, singular features may be formed by a shift of laminar projection origins (“externopyramidization”) in many cortical areas from lower to upper layers (Goulas et al., 2018; Sanides, 1962, 1970), a postnatal dendritic expansion of associative projection layer IIIc pyramidal cells, for example, in the prefrontal cortex (Petanjek et al., 2019), as well as more generally a prolonged period of postnatal development with considerable involvement of epigenetic processes of synapse selection and connectomic reorganization (Changeux, 2022; Changeux et al., 2021). We have suggested that these features may be controlled through specific gene regulatory events, although the concrete mechanisms are presently unknown.

Recent findings by several groups support and extend this hypothesis. For instance, Hendy et al. (2020) identified genes that are enriched specifically in the supragranular layers of the human cortex compared to mice. These genes show a relatively protracted expression in the human brain, corresponding to an extended duration of cortical connectivity development, and they may contribute to the characteristically expanded network of frontal cortico-cortical projections in the human brain. Moreover, Heyer et al. (2022) observed a selective expansion of supragranular layers II and III of left temporal cortex (specifically Brodmann area 21) in subjects with higher verbal and general IQ. This expansion was associated with larger cell body size of pyramidal neurons, which can support faster propagation of action potentials and may serve to improve information processing. Such observations directly confirm predictions of our connectomic hypothesis and demonstrate that variations of gene expression, particularly in upper cortical layers, may have affected developmental trajectories of the human brain, resulting in characteristically expanded and modified connectivity supporting enhanced cognitive functioning.

## A NATURAL AXIS OF OUTSIDE-TO-INSIDE CORTICAL ORGANIZATION

In addition to the features mentioned above, there is another fundamental aspect of the organization of mammalian brains in general, and the human cerebral cortex in particular, the alignment of several structural and functional features along a principal gradient, or *natural axis*, of cortical organization (Goulas et al., 2019, 2021; Sydnor et al., 2021).

**Figure 1.**
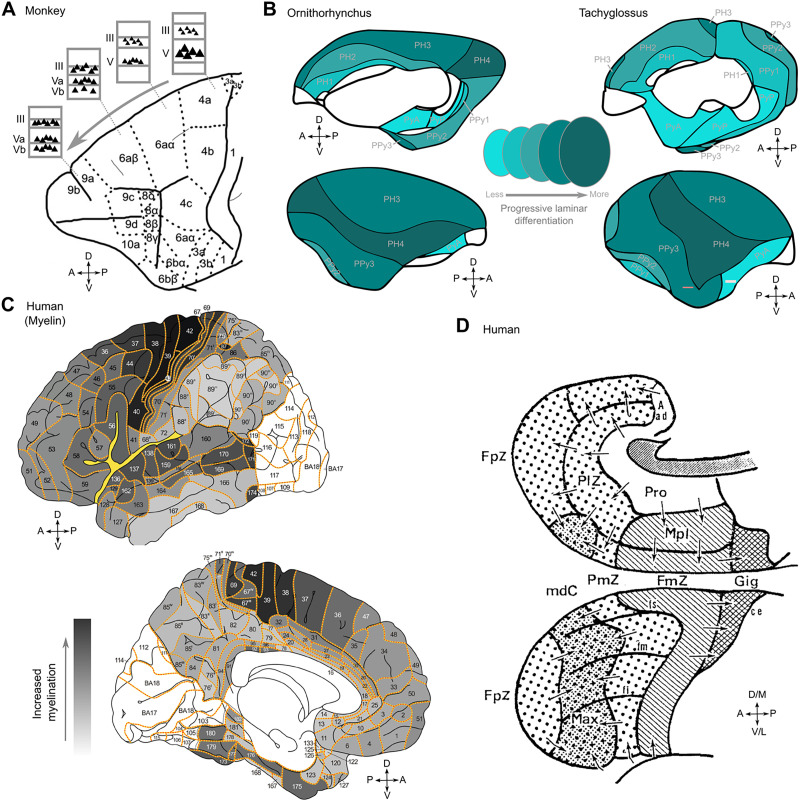
Gradation principle of the cerebral cortex of human and nonhuman animals. (A) Gradation along the caudal to rostral axis in the monkey frontal cortex based on its cytoarchitecture (Vogt & Vogt, 1919) showing a shift of large pyramidal cells from the deep to the upper cortical layers. Note that the very large projection neurons in deep layer V of primary motor cortex, area 4, are not necessarily an indication of the “inside” nature of this area (Barbas & García-Cabezas, 2015; Shipp, 2005). Apart from these Betz cells, providing direct and strong output to the spinal cord, the largest pyramidal neuron bodies in area 4 are located in layer IIIc (García-Cabezas et al., 2020). (B) Cytoarchitecture-based, whole-cortex gradients in two monotremes, ornithorynchus and tachyglossus (Abbie, 1940). (C) Cytoarchitectonic gradients in the human frontal lobe (Sanides, 1970). (D) Myeloarchitectonic gradients in the human cerebral cortex. Visual rendering from Nieuwenhuys and Broere (2017) using observations from Hopf (1955, 1956) and Hopf and Vitzthum (1957). Darker shading indicates stronger myelination.

The heterogeneity of the structure and function of the cerebral cortex was already appreciated in classical studies (e.g., Gall & Spurzheim, 1810; Broca, 1861). At least since the cytoarchitectonic studies of von Economo and Koskinas (von Economo, 1927; von Economo & Koskinas, 1925), who described an ordered system of structural cortical types, it has become clear that this structural heterogeneity is not arranged randomly or in a mosaic fashion, but organized along spatial gradients of the cerebral cortex. Examples of graded features include the laminar differentiation (i.e., how well different cortical layers, and particularly the granular layer IV, can be observed in a cortical region), the density of cells and the distribution of different cell types and morphologies across cortical layers (Dombrowski et al., 2001; Goulas et al., 2018), the degree of myelination, as well as fine-grained cellular morphology (Elston, 2002), forming a graded spectrum of cortical structural organization (John et al., 2021) (see Figure 1).

Later studies unified the concept of cortical gradients, by demonstrating that multiple such graded features may be spatially aligned. An important insight was the observation by Pandya and Sanides (1973) that cytoarchitectonic similarity and connectivity may be associated. This idea was formalized and expanded into the so-called Structural Model of Connections by Barbas and colleagues, which summarizes how the existence and laminar projection patterns of connections are aligned with the graded cytoarchitecture of primate cortical regions (Barbas, 1986; Barbas & Rempel-Clower, 1997; García-Cabezas et al., 2019). A further conceptualization of these fundamental interrelations, the Architectonic Type Principle (Hilgetag et al., 2019), also describes how microscopic morphological features of neurons as well as macroscopic features of cortical connectivity and topology are related to the structural types of cortical areas, as captured by their neuron density and laminar differentiation, and how these interrelations may arise from the ordered spatial-temporal ontogeny of the cerebral cortex (Beul et al., 2018; Beul & Hilgetag, 2020). Therefore, the spatially ordered gradient of structural types of cortical areas has emerged as a fundamental feature to which many other microscopic and macroscopic features of cortical organization are related, presumably organized by interactions during joined development. This includes patterns of gene expression; gross morphological features, such as cortical thickness and areal expansion during postnatal development; or functional aspects such as energy consumption or the time scale or complexity of physiological responses (Huntenburg et al., 2018; Murray et al., 2014; Sydnor et al., 2021; Wagstyl et al., 2015). Recently, we demonstrated that this gradient of structural cortical types is also aligned with the organization of different neurotransmitter receptors (Zilles & Palomero-Gallagher, 2017), forming a unified natural cortical axis (Goulas et al., 2021).

**Figure 2.**
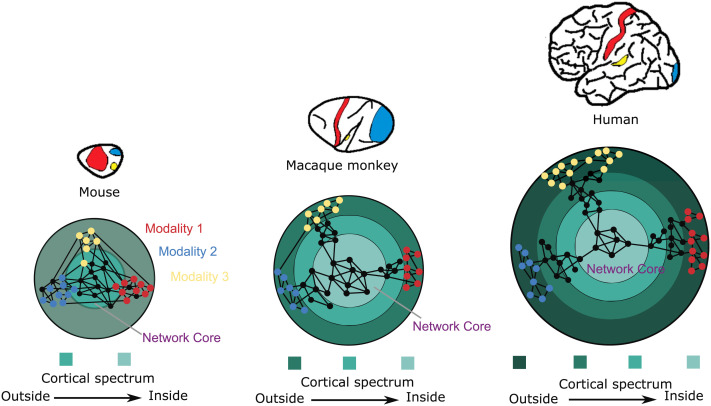
A multidimensional axis of mammalian and human cortical cytoarchitecture and connectivity. Cortical areas of the human brain are arranged by their cytoarchitectonic spectrum, from dense areas on the outside to more sparsely populated areas on the inside. These cellular densities are well correlated with other macroscopic and microscopic morphological features of cortical areas, such as laminar differentiation, cortical thickness, soma size, and spine density of pyramidal neurons (e.g., Beul & Hilgetag, 2019; John et al., 2021; van den Heuvel et al., 2015); compare Table 1. Moreover, as depicted in the lower panel, the existence or absence of connections is related to the similarity of cellular densities of the areas, as described by the Structural Model of Connections and Architectonic Type Principle (García-Cabezas et al., 2019; Hilgetag et al., 2019), with connections predominantly linking areas of a similar structure type, and the areas on the inside forming a densely connected core. The structural gradient also relates to the laminar organization of projection origins and terminations. Specifically, outside areas project to inside areas predominantly from the supragranular (upper) cortical layers. Conversely, inside areas project to outside areas predominantly from infragranular (deep) cortical layers, while areas of a similar type show a more balanced bilaminar pattern of projection origins. Compared to such arrangements for other mammalian species, such as the mouse and macaque monkey, the human arrangement possesses an expanded cytoarchitectonic gradient, as is apparent in the larger number of concentric circles than in other species (cf. Goulas et al., 2019; Hilgetag et al., 2019). Moreover, it shows a clear cytoarchitectonic differentiation between the core and the periphery of the cortical connectome. Figure adapted from Goulas et al. (2019), Changeux et al. (2021).

The axis can be considered natural in the sense that it does not strictly follow a prescribed spatial dimension, such as posterior to anterior. Instead, the axis has been proposed to stretch more generally from “sensory” to “association” or “transmodal” areas. A further conceptual simplification of this already simple picture would be to describe the axis as a progression from “outside” to “inside” areas of the cortex. Notably, the labels of outside versus inside areas are meant as a characterization of the endpoints of the axis, rather than as a dichotomous classification of all cortical areas into just two groups. Key features of outside versus inside areas are listed in Table 1, and a schematic depiction of the progressive arrangement of the cortical spectrum from outside to inside areas is given in Figure 2.

**Table 1.** Typical features of outside versus inside areas of the cerebral cortex

**Features**	**“Outside” areas**	**“Inside” areas**
*Cyto-architecture*	Dense (more cells), high laminar differentiation (eulaminate), larger pyramidal projection neurons in upper layers	Sparse (more neuropil), low laminar differentiation (agranular or dysgranular types), larger pyramidal projection neurons in deep layers
*Connectivity*	More locally connected, projections originating mostly from upper layers	Widely connected, projections originating mostly from deep layers
*Neurotransmitter receptors*	Specific, more inhibitory, ionotropic	Diverse, more excitable, metabotropic
*Maturation*	Late prenatal formation, early myelination, truncated plasticity	Early prenatal formation, late myelination, prolonged plasticity
*Functions*	Sensorimotor representations, high spatiotemporal resolution	Abstract representations, coarse spatiotemporal resolution

*Note*. These descriptions reflect characteristic features at the extreme end points of the cortical spectrum. Intermediate areas possess the features in a more graded manner.

The concentric arrangement in Figure 2 is based on a gradient of neural density and cytoarchitecture, which is fundamental for other structural features of mammalian organization, such as connectivity profiles, cellular morphological features, or cortical thickness (Beul & Hilgetag, 2019). As this structural spectrum closely aligns with several other structural, connectional, and functional aspects of cortical organization, the diagram effectively unifies many of the different kinds of cortical connection “hierarchies” described by Hilgetag and Goulas (2020), including feature gradients, projection sequences, orderings of directed “feedforward” and “feedback” projections, as well as hierarchical modular connectivity. The latter feature is represented by the core network module formed by the inside areas, which possess many long-distance projections. The present diagram also aligns with the sequence of synaptic steps from the outside to the inside of the brain, similar to the influential diagram of Mesulam (1998), providing an intuitive yet multidimensional representation of the natural axis of external to internal cortical organization.

It is important to note that the alignment between the various structural and functional feature gradients is not perfect. Indeed, Sydnor et al. (2021) presented associations among 10 cortical features, including tissue histology, evolutionary expansion, metabolism, gene expression, and function, that were widely differing in strength. Disagreements between feature gradients may arise from experimental noise, the linkage of features through indirect mechanisms, or unknown common factors rather than by direct relations, from misinterpretation, or by actual divergence, such as suggested by Paquola et al. (2019) for the increasing divergence between histological and functional gradients in transmodal cortices.

Thus, we do not think that all these cortical features are synonymous or directly related. A better understanding of their interrelations will be achieved by investigating and establishing the actual mechanisms that link the different aspects. Of particular relevance in this context is research into principles of cortical development and plasticity that will have to clarify how these features are mechanistically related, and will thus move beyond correlational studies.

## THE NATURAL AXIS OF THE HUMAN BRAIN

A natural axis of organization is present to a varying extent across the cerebral cortex of different mammalian species (Goulas et al., 2019). The natural axis of the human brain differs from that of other species in that it is expanded toward the outside, and these expanded outside regions possess a more elaborate cytoarchitecture and projection systems specifically in the upper cortical layers (Figure 2).

Compared to other primate species, the human brain forms along a developmental trajectory of prolonged pre- and postnatal development. This results in an expansion of late-developing structures, which concerns mostly “outside” areas of the dorso-lateral surface (Hill et al., 2010), as well as their late-developing laminar compartments, that is, particularly their upper layers. These areas and layers become large and very densely populated with neurons, also leading to a high laminar differentiation. Due to the association of these fundamental architectonic features with other structural, connectional, and functional aspects along the natural axis, the elongation of the human axis has direct consequences for the organization of the human connectome and ultimately human brain function.

Generally, human cortical organization becomes more multistaged and more differentiated at each of the stages. This also results in a deeper organization of cortical networks. Moreover, the expansion of outside areas is associated with a shift of large pyramidal projection neurons to the upper layers of these areas, forming an enhanced system for processing signals from the expanded sensory surface (Goulas et al., 2018). At the same time, outside areas become connectionally segregated from each other, due to expanded brain size and the increasing sparsity and localization of connections in the human brain (Changeux et al., 2021). Therefore, these regions are not directly connected with each other as they are in other species, but communicate with each other via the densely connected network core (Figure 2).

## FUNCTIONAL IMPLICATIONS

The concept of a unified cortical axis can be used to explain some of the special structural and functional features of the human brain. As a central feature, there is an expansion of this natural axis in the human brain due to prolonged brain development. This extension corresponds to the existence of further distinct stages, in terms of architecture, connectivity, and function, in the human brain compared to the brains of other species (cf. Hilgetag et al., 2019). Such an expansion of the cortical axis has a number of functional implications.

Foremost, due to the addition of areas, the human brain connectome possesses more processing stages, generally resulting in an increased depth of processing. Functionally, this likely results in increased representational depth, more accurate representations as well as more abstract representations at the stage of inside areas, similar to what is observed in artificial neural networks, particularly deep learning networks (LeCun et al., 2015; Wyss et al., 2006). There are also more specialized stages (due to the greater architectonic differentiation), which can form the basis of elaborate multiscale representations.

The elongation also results in greater separation of the different processing stages, and particularly in the separation of outside areas representing different sensory modalities, such as visual and auditory, that are no longer directly connected, but only via the network core of inside areas. This means that separate representations can be kept apart more stably, with less interference. The segregation implies reduced perturbation and greater stability of representations of the different sensory modalities, and may particularly support cognitive functions such as working memory (Rodriguez et al., 2019). The segregation is balanced by hierarchical modular connectivity that facilitates recursive integration and scaling (Moretti & Muñoz, 2013). In particular, the central modules in this hierarchical modular connectivity are formed by the widely connected network core of inside areas. The clear architectonic differentiation of the core-periphery network structure in the human brain likely provides the basis of specialized representations of the sensory and motor interface with the world that is being integrated through the core of inside areas.

The prolonged developmental expansion of the outside areas results in more detailed sensory representations as well as a more intricate motor interface, which is essential for increased dexterity and writing as well as speaking. In addition, these outside areas possess an elaborate system of enlarged projection neurons in the upper cortical layers that can serve to support fast sensorimotor interactions with the outside world as well as underlie higher cognitive functions such as language and intelligence (Heyer et al., 2022). These enhanced outside-to-inside projections are balanced by a system of long-range connectivity formed by the network core of inside areas, which is hypothesized to be engaged as an important anatomical component of the Global Neuronal Workspace for conscious access (Dehaene et al., 1998).

Further studies of this kind will need to determine the relevance of individual and combined connection features for brain function. In particular, computational studies may provide an avenue for isolating the influence of individual connectomic fundamentals and studying them in selected combinations in a more accessible way than is possible in experimental research.

In summary, the concept of the natural axis of human cortical organization provides a flexible framework for integrating a multitude of structural, developmental, and evolutionary aspects and relating them to the extraordinary cognitive abilities of the human brain.

## AUTHOR CONTRIBUTIONS

Claus C. Hilgetag: Conceptualization; Funding acquisition; Writing – original draft; Writing – review & editing. Alexandros Goulas: Conceptualization; Visualization; Writing – original draft; Writing – review & editing. Jean-Pierre Changeux: Conceptualization; Funding acquisition; Writing – original draft; Writing – review & editing.

## FUNDING INFORMATION

Claus C. Hilgetag, Human Brain Project, Award ID: SGA2, SGA3. Jean-Pierre Changeux, Human Brain Project, Award ID: SGA2, SGA3. Alexandros Goulas, Deutsche Forschungsgemeinschaft (https://dx.doi.org/10.13039/501100001659), Award ID: SPP 2041. Claus C. Hilgetag, Deutsche Forschungsgemeinschaft (https://dx.doi.org/10.13039/501100001659), Award ID: SPP 2041. Claus C. Hilgetag, Deutsche Forschungsgemeinschaft (https://dx.doi.org/10.13039/501100001659), Award ID: SFB 936/A1. Claus C. Hilgetag, Deutsche Forschungsgemeinschaft (https://dx.doi.org/10.13039/501100001659), Award ID: TRR 169/A2. Claus C. Hilgetag, Deutsche Forschungsgemeinschaft (https://dx.doi.org/10.13039/501100001659), Award ID: SFB 1461/A4.

## TECHNICAL TERMS

Connectomic fundamentals: Characteristic connectivity features underlying computational and functional properties of the brain.
The connectomic hypothesis: Suggests that the characteristic connectivity of the human brain is responsible for the extraordinary human cognitive abilities.
Sparsification: Decreasing density of brain networks as their size increases.
Modularity of connectivity: Arises when some regions are more frequently or densely connected with each other than with other regions of a network.
Core: Network regions that are particularly densely connected with each other, more than what would be expected by the general number of their connections.
Externopyramidization: The shift in the relative soma size of supragranular versus infragranular pyramidal neurons toward larger supragranular neurons.
Epigenetic processes: Phenotype modifications that do not involve alterations in the DNA sequence.
Supragranular: Cortical layers above the granular layer IV. Infragranular: cortical layers below the granular layer IV.
Deep learning networks: Artificial neuronal networks with more than one intermediate layer between inputs and outputs.
Global Neuronal Workspace: A distributed core-periphery network architecture for computations underlying conscious and unconscious cognitive functions.
